# A transcriptional landscape of 28 porcine tissues obtained by super deepSAGE sequencing

**DOI:** 10.1186/s12864-020-6628-7

**Published:** 2020-03-14

**Authors:** Tinghua Huang, Min Yang, Kaihui Dong, Mingjiang Xu, Jinhui Liu, Zhi Chen, Shijia Zhu, Wang Chen, Jun Yin, Kai Jin, Yu Deng, Zhou Guan, Xiali Huang, Jun Yang, Rongxun Han, Min Yao

**Affiliations:** College of Animal Science, Yangtze University, Jingzhou, 434025 Hubei China

**Keywords:** RUNX1, Super deepSAGE, PBMC, LPS

## Abstract

**Background:**

Gene expression regulators identified in transcriptome profiling experiments may serve as ideal targets for genetic manipulations in farm animals.

**Results:**

In this study, we developed a gene expression profile of 76,000+ unique transcripts for 224 porcine samples from 28 tissues collected from 32 animals using Super deepSAGE technology. Excellent sequencing depth was achieved for each multiplexed library, and replicated samples from the same tissues clustered together, demonstrating the high quality of Super deepSAGE data. Comparison with previous research indicated that our results not only have good reproducibility but also have greatly extended the coverage of the sample types as well as the number of genes. Clustering analysis revealed ten groups of genes showing distinct expression patterns among these samples. Our analysis of over-represented binding motifs identified 41 regulators, and we demonstrated a potential application of this dataset in infectious diseases and immune biology research by identifying an LPS-dependent transcription factor, runt-related transcription factor 1 (RUNX1), in peripheral blood mononuclear cells (PBMCs). The selected genes are specifically responsible for the transcription of toll-like receptor 2 (TLR2), lymphocyte-specific protein tyrosine kinase (LCK), and vav1 oncogene (VAV1), which belong to the T and B cell signaling pathways.

**Conclusions:**

The Super deepSAGE technology and tissue-differential expression profiles are valuable resources for investigating the porcine gene expression regulation. The identified RUNX1 target genes belong to the T and B cell signaling pathways, making them novel potential targets for the diagnosis and therapy of bacterial infections and other immune disorders.

## Background

The domestic pig (*Sus scrofa*) is an important animal farmed for meat worldwide and has been used as an alternative model for studying genetics, nutrition, and disease [1–3]. The swine research community has created a large database of the pig transcriptome [4]. The recently released pig genome sequence (*S. scrofa* 10.2) [5] and associated annotation greatly enhance our knowledge of pig biology [6, 7]. Currently, it is estimated that the porcine genome encodes for ∼20,000 genes [5]. Transcriptome analysis indicates that, of the total, actively transcribed genes represent only a mere fraction of 15,000 genes in all tissues [8]. Several research groups have created microarray transcriptome profiling data for humans [9, 10], mouse [11, 12], and rat tissues [13]. In the pig, several Expressed Sequence Tag (EST) sequencing projects, microarray platforms, longSAGE, and deep sequencing projects have developed gene expression profiles across a range of tissues [8, 14, 15]. In comparison to other model organisms, the pig transcriptome data has its limitations in terms of coverage of tissues and genes [4]. Here, we present Super deepSAGE (serial analysis of gene expression by deep sequencing) profiling data for pig tissues with wide gene coverage and annotation. Using the K-means clustering analysis and motif binding site enrichment analysis, we have identified key regulators for co-expressed genes. A detailed analysis of one such identified transcription factor, RUNX1, illustrates the impact of the data.

## Results and discussion

### Analysis of the complexity and diversity of super deepSAGE data across tissues

**Fig. 1:**
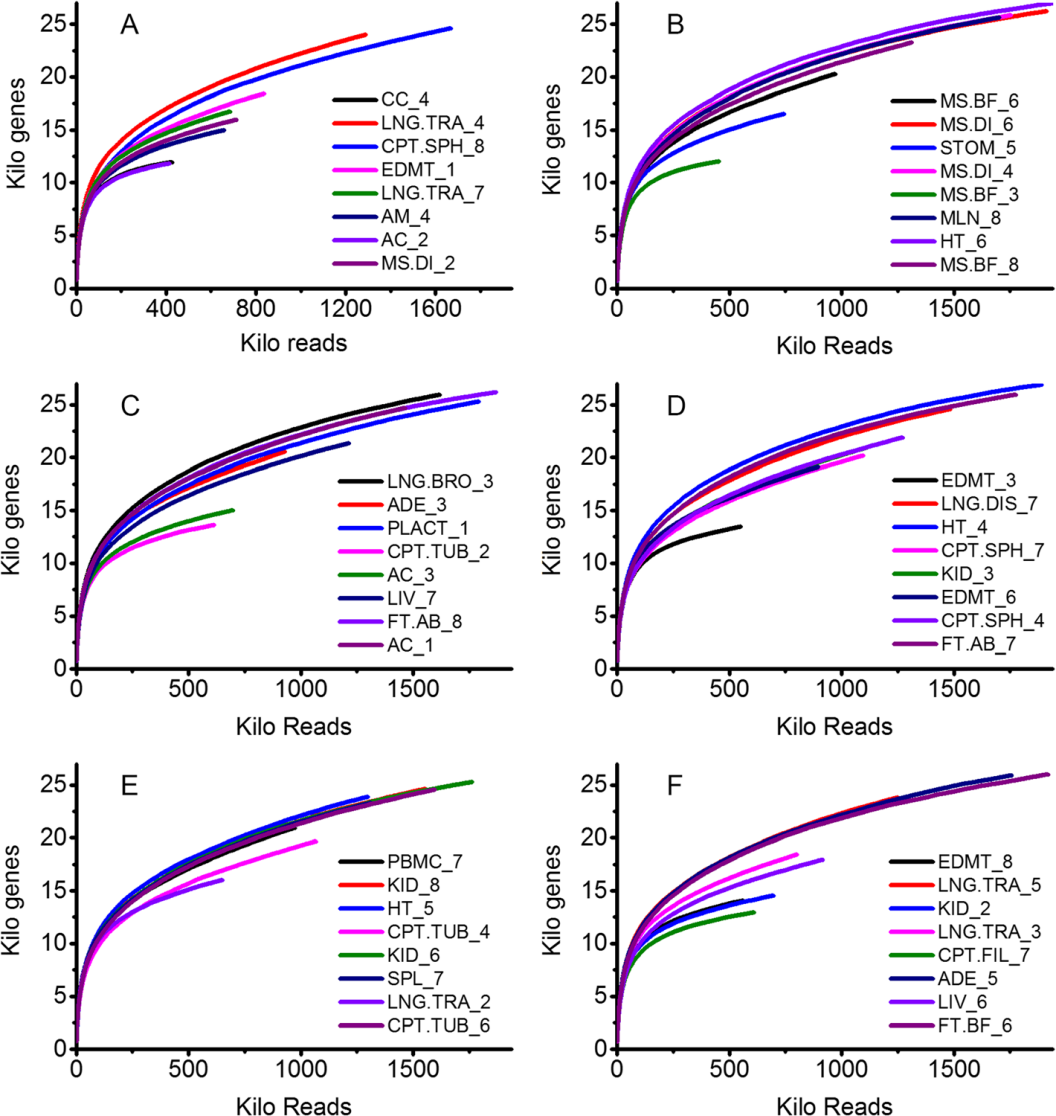
Rarefaction analysis of covered genes/transcripts in porcine tissues and cells Super deepSAGE library. Plot **a** to **f** shows the covered Kilo transcripts per Kilo reads in the first six Super deepSAGE sequencing runs. The samples in each sequencing run were randomized and detailed information is given in Table 1

Super deepSAGE obtained ~ 5 million reads per sample with an average sequencing depth of 71X (total number of genes identified by deep sequencing / total number of aligned reads, sequencing matrix is listed in Supplemental document 1). A total of 32,213 transcripts were covered by Super deepSAGE. Rarefaction analysis of a size-fractionated library for each tissue was performed to determine the complexity and diversity of pig tissues [16]. The sequencing depth achieved using eight samples-multiplexed deep sequencing technique (added different linker and pooled eight samples together to a single deep sequencing run) reached near-saturation of transcript discovery within all size ranges. Saturation was seen very early in Super deepSAGE sequencing data due to low tag complexity (number of tags) in libraries (Fig. 1a-f showed the first six deep sequencing runs). Samples from the same sequencing run were compared using reads from different size-fractionated libraries to further investigate the diversity of the relationship between sequencing depth and transcript discovery. In all deep sequencing runs, tissues exhibited transcriptome diversity in terms of both the total number of reads and the number of transcripts discovered. For example, the muscle tissue (MS.DI_2), saturated much sooner than the conceptus (CPT.SPH_8) and fewer transcripts were discovered in the first deep sequencing run (Fig. 1a). Similar sequencing depth and diversity were obtained using size-fractionated reads numbers from the other 22 sequencing run and discovered transcript numbers as outcome measures (Supplemental Fig. SA-D).

### Data quality and internal consistency control using principal component analysis (PCA)

**Fig. 2:**
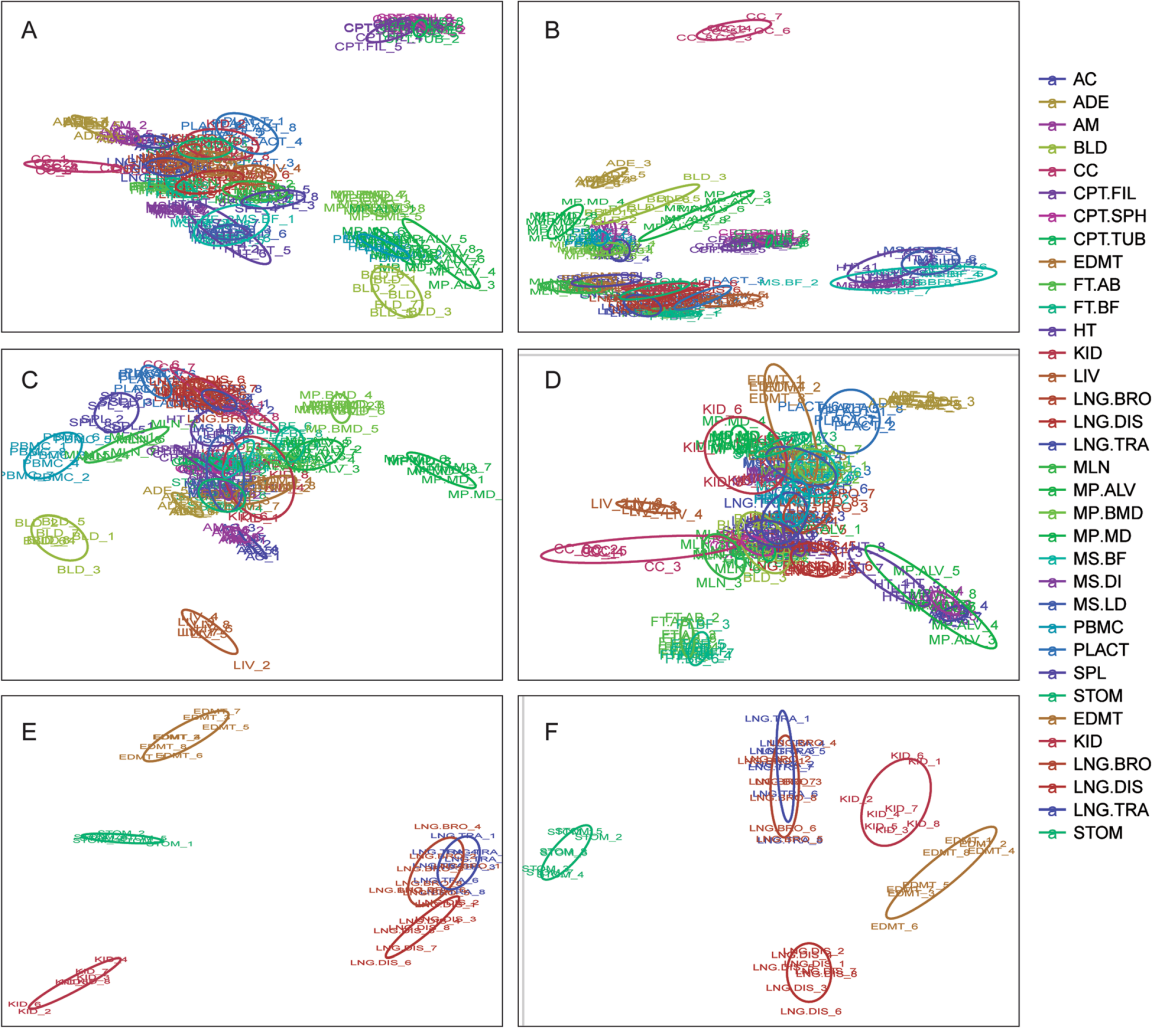
Principal component analysis of the Super deepSAGE sequencing data. **a**) to **d**) shows the top eight principal components of all 224 samples from the 28 tissues (two principal components per each plot). Samples separated in plot **a** to **d** were removed, and PCA was re-calculated with the remaining samples (fat, placenta, endometrium, kidney, lung, and stomach grouped). **e**) and **f**) shows the top four principal components of all the remaining samples (two principal components per each plot)

Principal component analysis (PCA) was used to check if the samples clustered together according to their tissue source [17]. Even though the samples were collected from 32 individual animals from different families, genders, and ages (Table 1), the PCA plot confirmed that the samples from the same tissues clustered together and were distinct from other samples (Fig. 2). The transcripts in conceptus, blood, and macrophages had relatively distinct expression profiles and segregation from the rest of the samples when plotted using the first two components of the PCA analysis (Fig. 2a). The adenohypophysis, cerebral cortex, heart, and muscle were aggregate and separated from other samples when plotted using the third and fourth components (Fig. 2b). The adrenal, liver, mesenteric lymph nodes, peripheral blood mononuclear cell, and spleen deviated from other samples when plotted using the fifth and sixth components (Fig. 2c). When eliminating those samples from the datasets and re-calculating the PCAs, the remaining samples; fat, placenta, endometrium, kidney, lung, and stomach grouped differently according to the tissue/cell types (Fig. 2d-f). Tissues having similar cellular composition and biological function, like alveolar and monocyte-derived macrophages or heart and skeletal muscles, clustered closely together but were distinct from each other.

**Table 1:** Detailed information of the collected samples

Code	Tissue	Code	Tissue
AC	Adrenal cortex	FT.BF	Back fat tissue
AM	Adrenal medulla	KID	Kidney
CPT.SPH	Conceptus spherical	ADE	Adenohypophysis
CPT.TUB	Conceptus tubular	MP.BMD	Bone-marrow derived macrophage
FT.AB	Abdominal fat tissue	MS.BF	Biceps femoris
MS.DI	Diaphragm muscle	EDMT	Endometrium
STOM	Stomach	BLD	Blood
CPT.FIL	Conceptus filamentous	PBMC	Peripheral blood mononuclear cell
MS.LD	Longissimus dorsi	HT	Heart
LNG.TRA	Lung porcine trachea	CC	Cerebral cortex
PLACT	Placenta	MP.MD	Monocyte derived macrophage
LNG.BRO	Lung porcine bronchus	MP.ALV	Porcine alveolar macrophages
LNG.DIS	Lung porcine distal	MLN	Mesenteric lymph nodes
SPL	Spleen	LIV	Liver

### Comparison of the super deepSAGE data with previously published microarray research

**Fig. 3:**
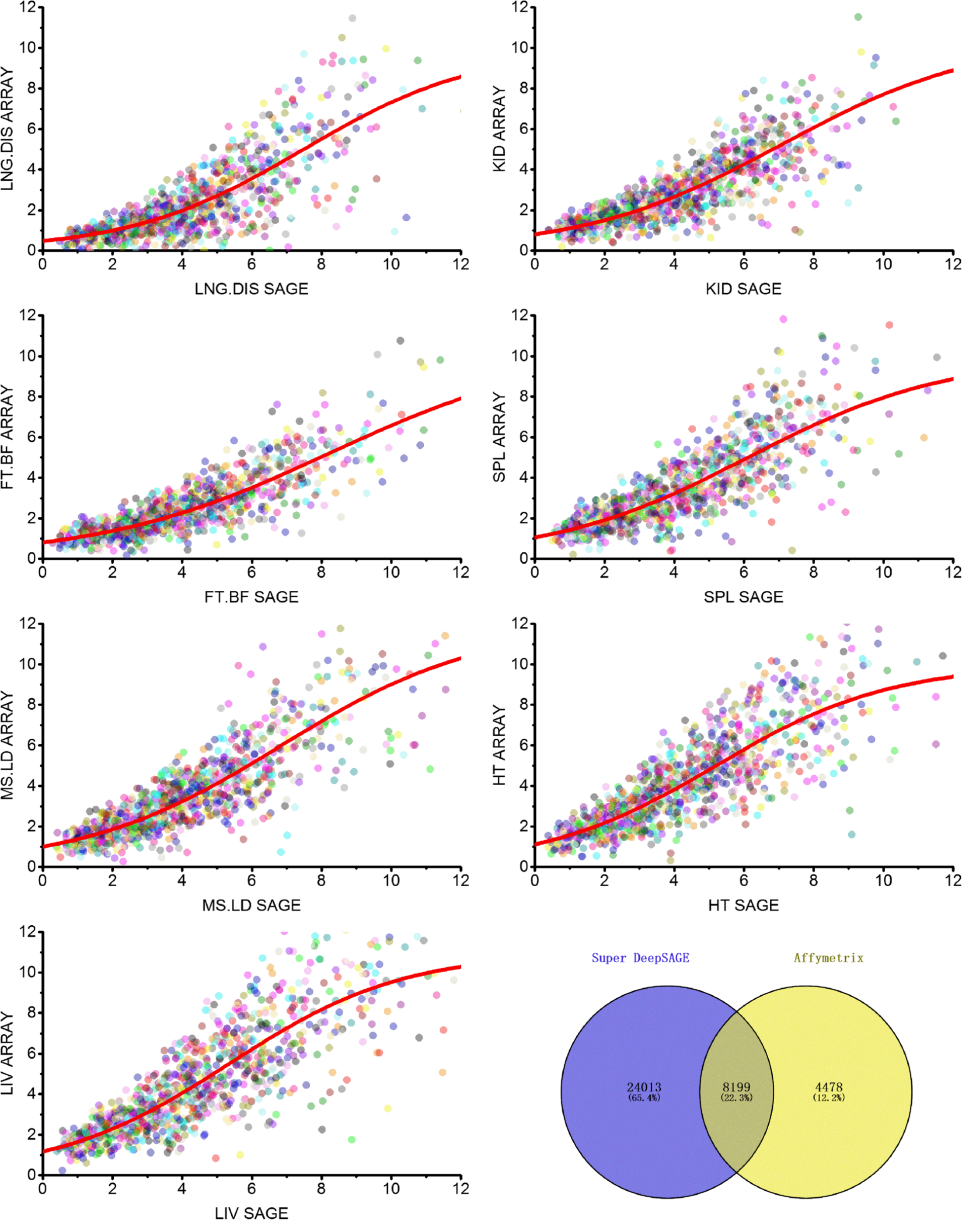
Comparison of the expression profiles of the 18,306 common transcripts between Super deepSAGE and microarray platforms. Scatter plots show the averages (between biological duplicates) of log2 transformed expression values of transcripts between two platforms. The relationship between the expression profiles generated in the two platforms is depicted as a smoothing spline (red)

The expression profiles were compared with previously published microarray data [8]. The processed microarray datasets were acquired from the GEO database and normalized to the Super deepSAGE data using the quantile normalization method to make these two datasets comparable. There is a total of 8199 common transcripts for seven tissues in both platforms, a total of 24,013 transcripts remain undetected by the Affymetrix platform, and a total of 4478 transcripts were undetected in Super deepSAGE experiments (Fig. 3). Among the commonly detected transcripts, a high correlation (r = 0.85–0.93 and *p*-values less than 1.0 × e^− 30^) was calculated between the gene expression profiles generated by the two platforms (Fig. 3). A similar dynamic range was observed in both platforms for transcripts with a relative expression level (log2 based and quantile normalized expression value) between 4.0 and 9.0. Differences in expression profiles were apparent between the two platforms as several genes exhibiting relatively higher or lower expression values in either platform deviated from the slope (Fig. 3). All transcripts had an expression value in the microarray due to background hybridization or noise, regardless of whether it was truly expressed or not. The overall dynamics of the fitted curve tend to show that the Super deepSAGE platform is a more sensitive technique than the microarray for low expression genes that show a concaved trend at the lower ends (with relative expression level less than 4.0 in Fig. 3). For those genes with high expression levels, variability is high in both Super deepSAGE and microarray platforms. In the seven overlapped tissues between Super deepSAGE and microarray, the 50 highest expressed Super deepSAGE tags, 38 (76%) found corresponding probe sets in the 50 highly expressed genes, and only three tags showed a statistically significant difference between Super deepSAGE and microarray data.

### Identification of tissue-differential expression of transcripts

**Fig. 4:**
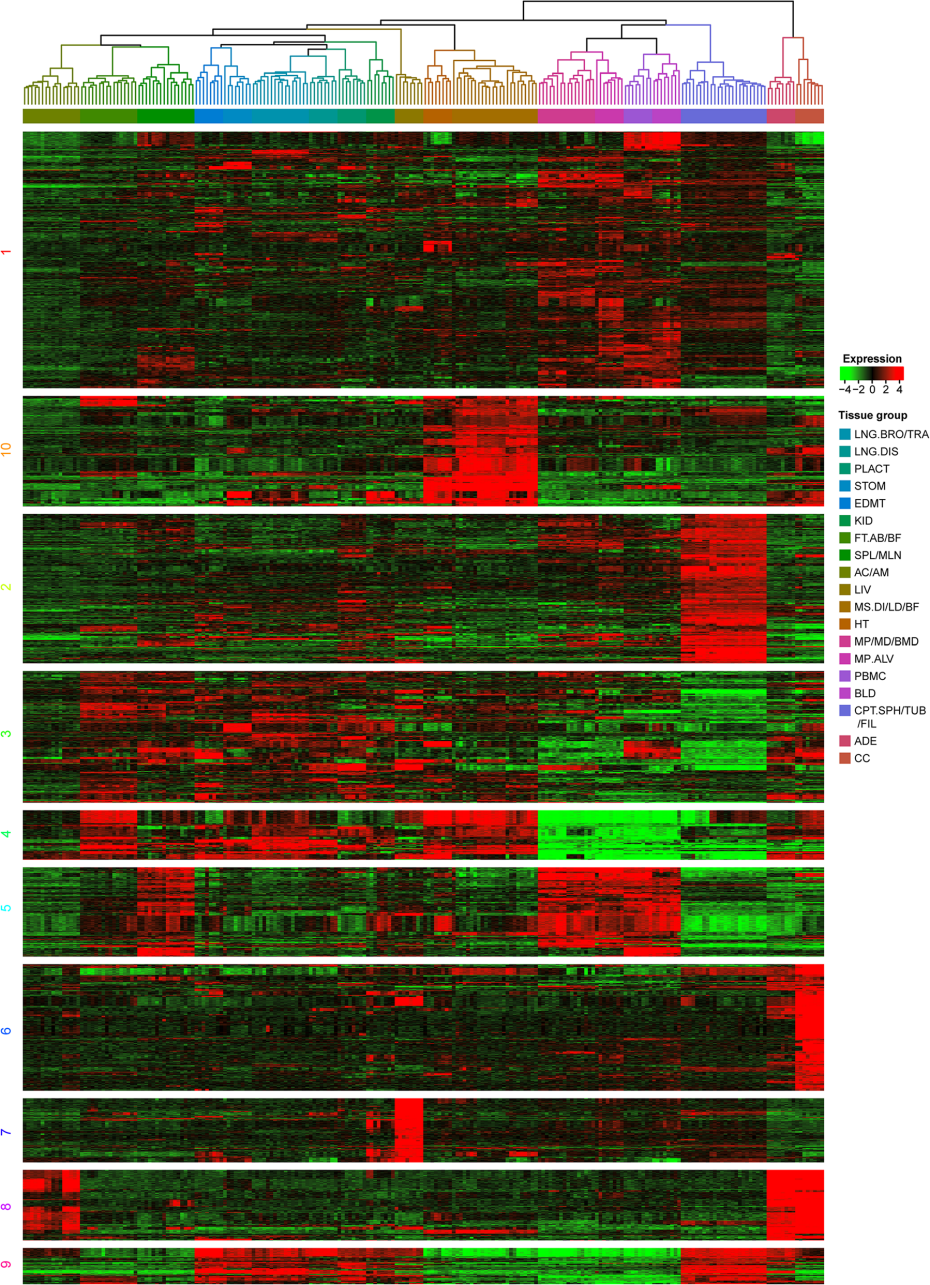
K-means clustering analysis of differentially expressed genes across tissues. Data adjustment (median center and normalization) was performed before the clustering analysis. The color codes of red, white, black, and dark green represent high, average, low, and absence of expression, respectively. A detailed view of expression pattern and internal structure of each gene cluster were constructed by hierarchical clustering and is shown in plot areas from 1–10

A total of 4165 transcripts showed significant up or down-regulation in at least one tissue, in comparison to the average tag count for 27 tissues. K-means clustering analysis was then performed by trying a different number of centers (K from 5 to 28) and several random sets (S from 10 to 1000). An ad hoc method comparing each tissue to the average tag count for all 27 tissues was performed, and a very stringent threshold was set (fold change > 5.0, *p*-value < 1.0 × 10^− 6^) to filter the tissues specifically expressing transcripts. We selected K = 10 and S = 400 to produce a clustered result with a clear expression pattern (by visualization), highly reproducible for each duplicated run (Fig. 4). The detailed clustering information is available in Supplemental document 2. The result indicated that Cluster 1 has the largest number of transcripts, and most of these transcripts were expressed at a low level in tissues, except macrophages, PBMCs, blood, and conceptus in which it was moderately expressed. The conceptus expressed transcripts were in Cluster 2, while the conceptus, macrophages, PBMCs, and blood down-expressed transcripts were in Cluster 4. The macrophages, PBMCs, blood, mesenteric lymph nodes, and spleen specific transcripts were in Cluster 5. The genes specifically expressed in the heart and skeletal muscles were in cluster 10. The cerebral cortex specific genes were in Cluster 6, and liver specifically expressed transcripts were in Cluster 7. The adrenal cortex, adrenal medulla, cerebral cortex, and adenohypophysis specific transcripts were in Cluster 8. Transcripts in Cluster 3 and Cluster 9 were ubiquitously expressed in multiple tissues.

### Identification of over-represented motif for tissues specifically expressed transcripts

The CLOVER software [18] with JASPAR PWM database [19] was used to identify over-represented transcription factor binding motifs for each gene cluster. The promoter regions for a transcript cluster (1000 bp upstream from the TSS) were determined using the Ensemble Biomart tool (*Sus scrofa* assembly 11.1, gene 99) [20]. The promoter regions for the transcripts detected, with a similar GC content, were used as background. Motifs having a *p*-value of ≤0.05 was significant (Table 2, top 5 motifs). The most significantly enriched motif in Class 1 is MZF1. TFAP2A and TFAP2C were also significantly enriched with a raw score higher than 30. In Class 2, there was only one significantly enriched motif, RHOXF1. In Class 3 and 4, there were five and four motifs with p-value < 0.05 respectively, but the raw score was lower than ten. In Class 5, there were at least five motifs with p-value < 0.05, and three of them, RUNX1, ASCL1, and Myod1 had a raw score higher than 30. In Class 6, the significantly enriched motifs with the highest score were SNAI2 and FIGLA, whereas, in Class 7, the significantly enriched motifs with the highest score was NR4A2. In Class 8, there was only one motif ZEB1 enriched in the promoter region of these transcripts. In Class 9, all the enriched motifs had a raw score of less than ten. In Class 10, the top three motifs were Ascl2, Myog, and Tcf12.

**Table 2:** Significantly over-represented binding motifs in the promoter region of transcripts showing a similar expression pattern

Gene cluster	Jaspar ID	TF name	Occurrence	Gene count	Raw score	FDR
Class 1	MA0056.1	MZF1	291	291	91.6	0
Class 1	MA0810.1	TFAP2A(var.2)	165	165	33.1	0.002
Class 1	MA0524.2	TFAP2C	149	149	32.3	0.003
Class 1	MA0811.1	TFAP2B	143	143	29.1	0.004
Class 1	MA0507.1	POU2F2	53	53	8.35	0.006
Class 2	MA0719.1	RHOXF1	142	142	6.96	0.008
Class 3	MA1105.1	GRHL2	28	28	6.99	0.003
Class 3	MA0842.1	NRL	60	60	3.67	0.006
Class 3	MA0164.1	Nr2e3	52	52	2.42	0.003
Class 3	MA0029.1	Mecom	23	23	2.25	0.009
Class 3	MA0117.2	Mafb	49	49	1.69	0.004
Class 4	MA0691.1	TFAP4	20	20	4.93	0.004
Class 4	MA0091.1	TAL1::TCF3	16	16	2.43	0.006
Class 4	MA0616.1	Hes2	19	19	0.8	0.003
Class 4	MA0089.1	MAFG::NFE2L1	21	21	−1.09	0.005
Class 5	MA0002.2	RUNX1	135	135	55.5	0
Class 5	MA1100.1	ASCL1	79	79	46.8	0.008
Class 5	MA0499.1	Myod1	59	59	31.6	0.003
Class 5	MA1124.1	ZNF24	30	30	18.6	0.002
Class 5	MA1109.1	NEUROD1	57	57	9.76	0.004
Class 6	MA0745.1	SNAI2	75	75	18.7	0.001
Class 6	MA0820.1	FIGLA	77	77	17.6	0.003
Class 6	MA0138.2	REST	6	6	7.13	0.002
Class 6	MA0665.1	MSC	36	36	6.63	0.002
Class 6	MA0691.1	TFAP4	30	30	5.79	0.003
Class 7	MA0160.1	NR4A2	59	59	15.5	0
Class 7	MA0693.2	VDR	31	31	4.29	0.004
Class 7	MA0017.2	NR2F1	27	27	4.12	0.009
Class 7	MA1142.1	FOSL1::JUND	38	38	3.25	0.001
Class 7	MA0059.1	MAX::MYC	16	16	1.48	0.008
Class 8	MA0103.3	ZEB1	21	21	10.9	0.002
Class 9	MA0084.1	SRY	22	22	9.59	0.01
Class 9	MA0130.1	ZNF354C	35	35	9.47	0.007
Class 9	MA0463.1	Bcl6	21	21	7.12	0.007
Class 9	MA0799.1	RFX4	7	7	5.64	0.007
Class 9	MA0798.1	RFX3	7	7	5.38	0.009
Class 10	MA0816.1	Ascl2	66	66	28.6	0.009
Class 10	MA0500.1	Myog	58	58	28.5	0.01
Class 10	MA0521.1	Tcf12	57	57	27.2	0.008
Class 10	MA0665.1	MSC	36	36	9.17	0.002
Class 10	MA0108.2	TBP	57	57	4.39	0.007

### Case report: confirmation of the regulatory roles of RUNX1 in PBMCs in pig

In the cluster heatmap (Fig. 4), Class 1 and 5 tentatively show (by visualization) high expression in macrophages, PBMCs, and blood. However, the expression level of genes in Class 1 was lower than in Class 5. Further, mesenteric lymph nodes and spleen specific transcripts in Cluster 5 indicated that this class is an immunity-related gene cluster. The top over-represented motif in Class 5 is RUNX1, and literature search of its targets indicated that TLR-2 (Toll-like receptor), LCK (tyrosine kinases), and VAV1 (Rho family GTPases) play a role in T and B-cell development and activation. These three representative RUNX1 targets were selected for further experimental validation.

### Confirmation of the RUNX1 binding site in the promoter region of TLR-2, LCK, and VAV1

**Fig. 5:**
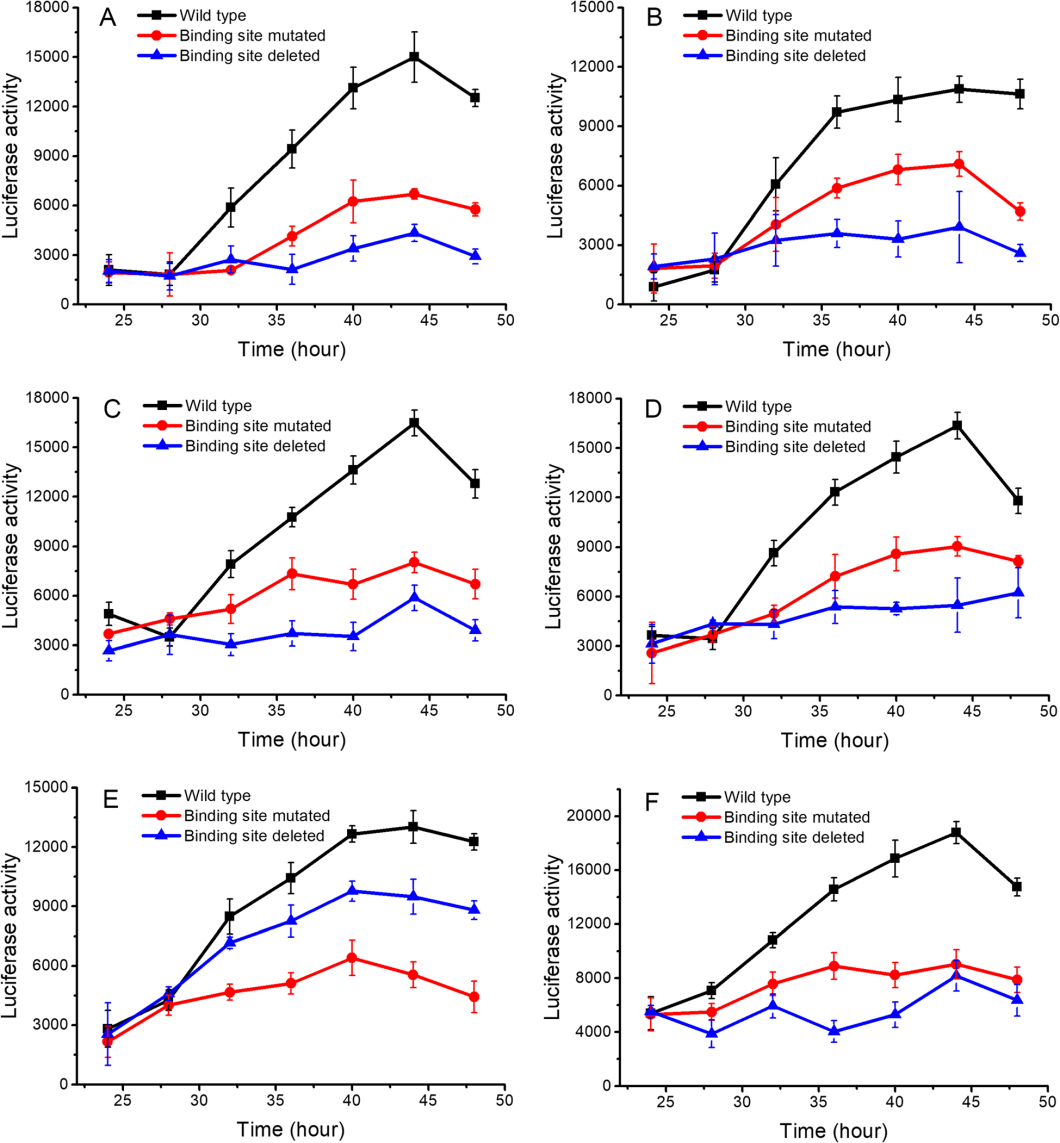
Luciferase reporter assay of the RUNX1 targets. One wild-type promoter construct (containing the predicted RUNX1 binding site), two mutant constructs (mutated or not containing the binding site) were investigated. The mutant construct (black) was identical to the wild-type, except that the RUNX1 binding site was deleted or mutated. The line graphs show the luciferase activity after the reporter plasmids were transfected into PBMCs (**a**-**c**) or macrophages (**d**-**f**). Three RUNX1 target genes have been investigated (**a** and **d**: TLR-2, **b** and **e**: LCK, **c** and **f**: VAV1). The error bars represent the mean ± standard deviation of three duplicate sample sets

The toll-like receptor 2 (TLR-2), lymphocyte-specific protein tyrosine kinase (LCK), and vav1 oncogene (VAV1) plasmid containing the 1Kb putative promoter sequence were used in in vivo studies (wild type). To show the regulatory effect of RUNX1, the binding site of RUNX1 in TLR-2, LCK, and VAV1 was mutated or deleted. Reporter vectors constructed by the wild type, mutated, or deleted promoter sequences were transfected into the peripheral blood mononuclear cells (PBMCs), and luciferase activity was monitored. Binding site deletion significantly attenuated the expression of the downstream reporter luciferase activity (*p* < 0.05), indicating that RUNX1 could interact with the target site and regulate the expression of the downstream reporter gene (Fig. 5a-c). The mutated vectors showed significant attenuation of the activity of downstream luciferase at 40, 44, and 48 h post-transfection (p < 0.05) indicating a regulatory relationship between RUNX1 and the targets. Another experiment was performed using mouse macrophage cells (RAW 264.7) to validate the hypothesis further. Consistent with the previous results, mutation of the RUNX1 binding sites in TLR-2, LCK, and VAV1 promoter sequence significantly attenuated the activity of downstream luciferase at 40, 44, and 48 h post-transfection (Fig. 5d-f). The luciferase reporter activity after transfection with the wild-type vector was significantly higher in macrophage cells than in the PBMC assays, suggesting that the endogenous RUNX1 expression in mouse macrophage cells was higher than in PBMCs.

### RNA flow cytometry analysis of RUNX1 targets in LPS and RUNX1 inhibitor-treated PBMCs

**Fig. 6:**
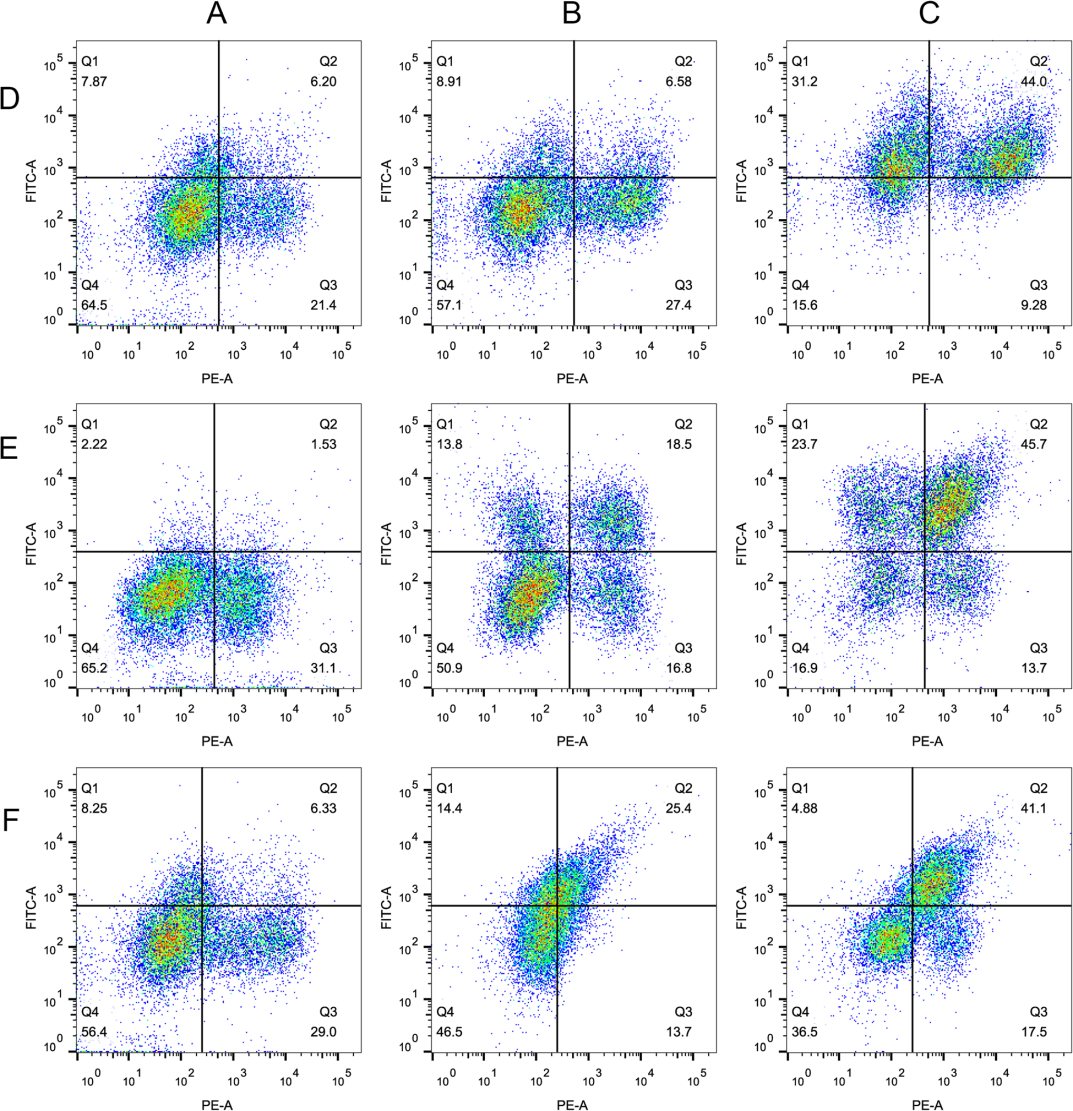
Simultaneous staining of the target gene and CD14 protein in rested and stimulated PBMCs. Plots of PBMCs that were left untreated (**a**) or were stimulated with LPS plus RUNX1 inhibitor for 6 hours (**b**) or were stimulated with LPS only (**c**) and labeled with antibodies that bind to CD14 (PE-A) and target protein (FITC-A, **d**-**f**)

To show the effect of RUNX1 on three targets; TLR2, LCK, and VAV1, pig PBMCs were stimulated with LPS and/or RUNX1 inhibitor, for 6 h, during which their TLR2, LCK, VAV1, CD14 protein levels were monitored. Two subsets of cells readily emerged from CD14/TLR2 analysis in PBMCs: a CD14^hi^/TLR2^lo^ (CD14^high^/TLR2^low^) and a CD14^lo^/TLR2^lo^ population (Fig. 6d). The percentage of CD14^hi^/TLR2^lo^ cells increased in LPS plus RUNX1 inhibitor-treated samples, but the proportion of CD14^lo^/TLR2^lo^ cells remained unchanged. The percentages of TLR2^hi^ (for both CD14^hi^ and CD14^lo^) cells increased seven-fold in LPS alone treated samples compared with the non-treated controls. Four subsets of cells readily emerged from CD14/LCK analysis in PBMCs treated with LPS or RUNX1 inhibitor: a CD14^hi^/LCK^lo^, CD14^hi^/LCK^hi^, CD14^lo^/LCK^hi^, and CD14^lo^/LCK^lo^ population (Fig. 6e). The percentage of CD14^hi^/LCK^hi^, and CD14^lo^/LCK^hi^ cells increased in LPS plus RUNX1 inhibitor-treated samples, and the proportion of CD14^hi^/LCK^lo^ cells was decreased. The percentage of CD14^hi^/LCK^hi^ cells increased by 40% in LPS alone treated samples compared with the non-treated controls. Two subsets of cells readily emerged from CD14/VAV1 analysis in PBMCs: a CD14^hi^/VAV1^lo^ and a CD14^lo^/VAV1^lo^ population (Fig. 6f). The percentage of VAV1^hi^ (for both CD14^hi^ and CD14^lo^) cells increased four-fold in LPS plus RUNX1 inhibitor-treated samples. The percentages of VAV1^hi^ (for both CD14^hi^ and CD14^lo^) cells increased seven-fold in LPS alone treated samples compared with the non-treated controls and is two-fold higher than in LPS plus RUNX1 inhibitor-treated samples.

### Real-time PCR analysis of RUNX1 targets in LPS and RUNX1 inhibitor-treated PBMCs

**Fig. 7:**
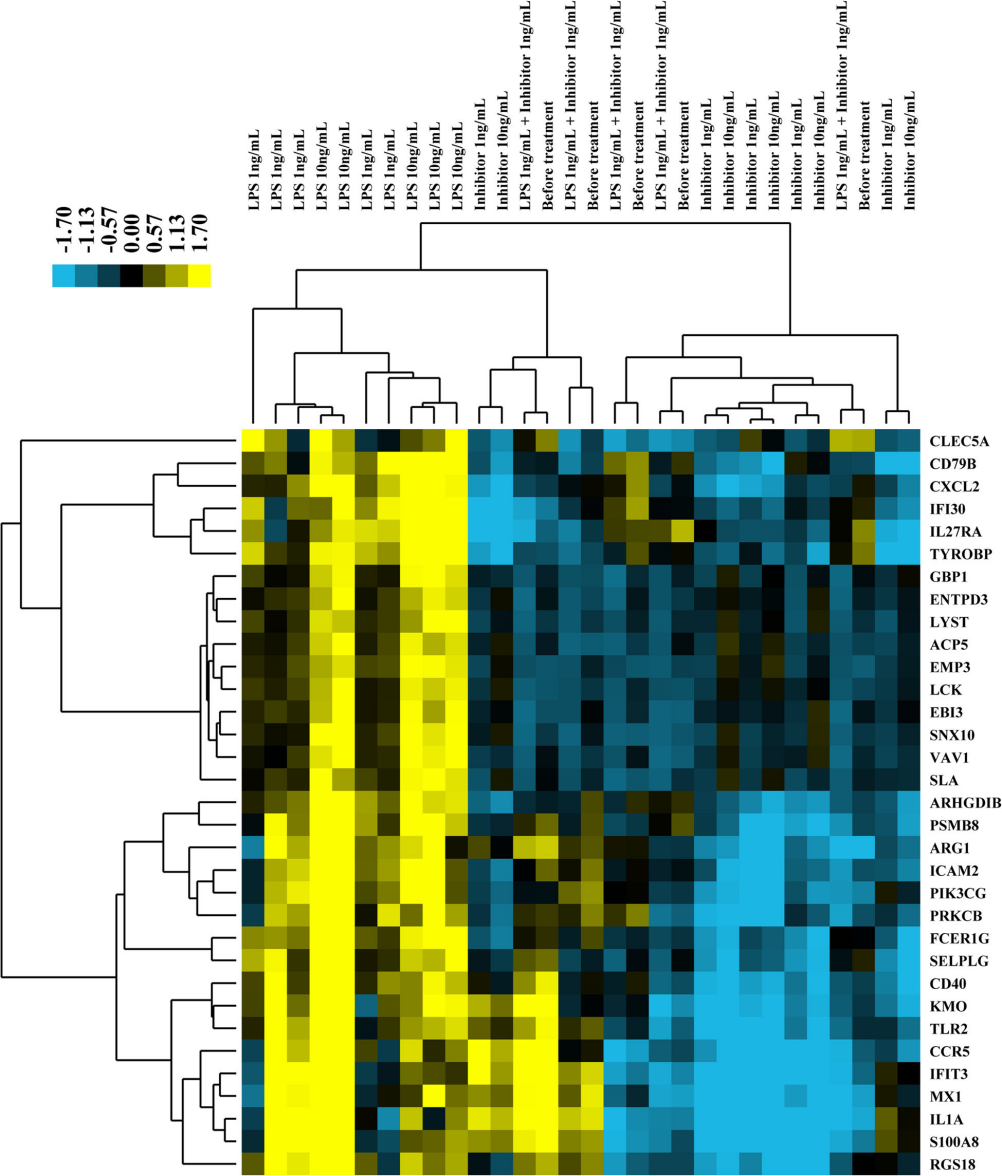
RUNX1 target gene expression in PBMCs treated with LPS or RUNX1 inhibitor. Cells were treated in vitro with two different doses of LPS (1 ng/ml, 10 ng/ml) and RUNX1 inhibitor (1 ng/ml, 10 ng/ml). Color codes of yellow, black, and blue represent expression levels of high, average, and low, respectively, across the treatments shown

To investigate if the expression patterns of the 33 RUNX1 target genes that have RUNX1 binding site (from Class 5, Supplemental document 3) could be modeled by LPS and RUNX1 inhibitor treatment in vitro, we performed real-time PCR assays for 33 genes after treating PBMCs (collected from a separate set of animals) with two different doses of LPS (1 ng/mL, 10 ng/mL), and RUNX1 inhibitor (1 ng/mL, 10 ng/mL). Samples were collected six hours post-stimulation. A total of 21 genes were induced significantly in response to at least one dose of LPS stimulation, as expression levels for these genes were different when compared to non-stimulated control. A total of 10 genes were down-regulated significantly in response to the RUNX1 inhibitor treatment. Hierarchical clustering analysis was used to determine whether the response of LPS stimulation was similar to the patterns detected in RUNX1 inhibitor treatment, and if any differences were observed depending on the dosage of LPS and RUNX1 inhibitor used. As shown in Fig. 7, the genes were grouped into two large clusters. In the upper part of the cluster (from CLEC5A to SLA) the expression patterns of samples with RUNX1 inhibitor treatment, RUNX1 inhibitor plus LPS treatment, and non-simulated controls tend to be similar. Different doses of the RUNX1 inhibitor did not affect the samples, as observed by the overlap of respective samples in the heatmap. The LPS treated samples were unique and were distinct from the RUNX1 inhibitor-treated groups and control groups. Similar to the RUNX1 inhibitor, different doses of LPS also did not affect the samples. The expression patterns of RUNX1 inhibitor plus LPS treatment samples tend to be similar to controls and RUNX1 treatment only samples because of the overlap in the heatmap. In the lower part of the cluster (from ARHGDIB to RGS18), the expression patterns of some of the samples following RUNX1 inhibitor treatment, RUNX1 inhibitor plus LPS treatment, and non-simulated controls tend to be similar with the LPS treated samples. The reason for this discrepancy could be due to variation in preparing the PBMCs and the stimulation process which are difficult parameters to control.

## Discussion

This study created transcriptomic datasets by Super deepSAGE using a large number of samples and a large number of biological repeats for the same tissue, and detected a large number of transcripts. To our knowledge, our work represents the largest tissue dataset profiled in a single study. We first analyzed the homogeneity of transcriptional information within samples of the same organ using PCA. Without prior information, we showed that samples from the same organ but different donors clustered together. This result is remarkable given the potential variability that could have been introduced for each sample with respect to the different animals. To analyze of similarities and differences between the expression profiles for different tissues, we used hierarchical clustering analysis. The dendrogram generated by this analysis reflected functional relationships between tissues. Our results also showed that gene expression profiling can distinguish each of the tissues.

The data demonstrated that tissue-differentially expressed genes can be distinguished to gene clusters which share similar expression profiles and those genes were co-regulated by common regulator genes. We found that genes in gene clusters were always expressed in tissue-specific manner. These findings are consistent with and strengthen the rationale for transcription rules for mammalian that transcription of genes turned on when and only when they were required. The transcription factors interact with the DNA recognition motifs, and regulate transcription of a large number of genes, and plays an important role in the regulation of tissue-differentially expressed genes. To interpret and understand gene regulation from the Super deepSAGE data obtained in this study, identifying the over-represented or under-represented motifs in the sequence showing similar expression patterns and factors binding to them, is necessary. Over-represented motifs were identified to play a regulatory role in the sequences, while under-representation indicated that the motif would have a harmful dis-regulatory effect.

RNA-seq can easily provide a much larger yield, have a large dynamic range and identify a larger number of genes and transcripts. However, literature shows limited number of studies using RNA-seq technology have been accomplished that run in parallel for a wide range of porcine tissues. The Super deepSAGE reduced cost by multiplexing and obtained data with good quality in terms of sequencing depth, gene coverage, and reproducibility. Few discrepancies were observed when comparing Super deepSAGE data with published microarray data. We predict two possibilities that could cause such discrepancies: 1) the SAGE tag was derived from two or more different transcripts, which were differentially expressed in the samples tested, and 2) the microarray probe set can target two or more transcripts due to sequence similarity. For example, the transcripts from the same gene family will always produce the same SAGE tag (attributable to the lower resolution power of Super deepSAGE) and preferred to hybridize to the same microarray probe set (can be minimized by design probe sets in the non-conserved region). Regardless of the discrepancies, we conclude that Super deepSAGE data is overall compatible with the microarray data and provide reliable gene expression profiles.

Among the transcription factors identified for tissue-differentially expressed genes, RUNX1 is a master regulator of hematopoiesis and plays a vital role in T and B cell development. RUNX1 is critical in induction of the immune cells, such as interleukin-2 (IL-2, [21], IL-3 [22], colony-stimulating factor 1 receptor (CSF1R, [23], CSF2 [24], and cluster of differentiation 4 (CD4), [25]. However, its roles in LPS-mediated inflammation in PBMCs remains unclear. In this study, regulations of TLR-2, LCK, and VAV1 have been confirmed by flow cytometry. TLR2 is an essential receptor for the recognition of a variety of pathogen-associated molecular patterns (PAMPs) from Gram-positive bacteria, including bacterial lipoproteins, lipomannan, and lipoteichoic acids [26]. LCK encoded protein is a key signaling molecule in the selection and maturation of developing T-cells [27]. The VAV1 encoded protein is important in hematopoiesis, playing a role in both T-cell and B-cell development and activation [28, 29]. These results suggest that RUNX1 might be a new potential target for resolving inadequate or uncontrolled inflammation in PBMCs.

## Conclusions

Gene expression analysis is extensively applied in the understanding of the molecular mechanisms underlying a wide range of biological processes such as host-pathogen interactions. Our dataset (of transcript levels in tissues) can serve as a reference dataset for comparison of expression analysis to detect aberrations in transcript levels of various biological functions. Therefore, the major focus of this manuscript was to demonstrate the biological importance of these profiles. We report that > 40% of the measured transcripts were differentially expressed between different tissues. We show that statistically the transcripts were co-regulated by a few important transcription factors. Our study led to the identification of key transcription factors that regulate gene expression in PBMCs. This data will improve the annotation of the pig genome, support biological studies and increase the utility of the pig as a meat source and model in medical research.

## Methods

### Development of super deepSAGE technology

**Fig. 8:**
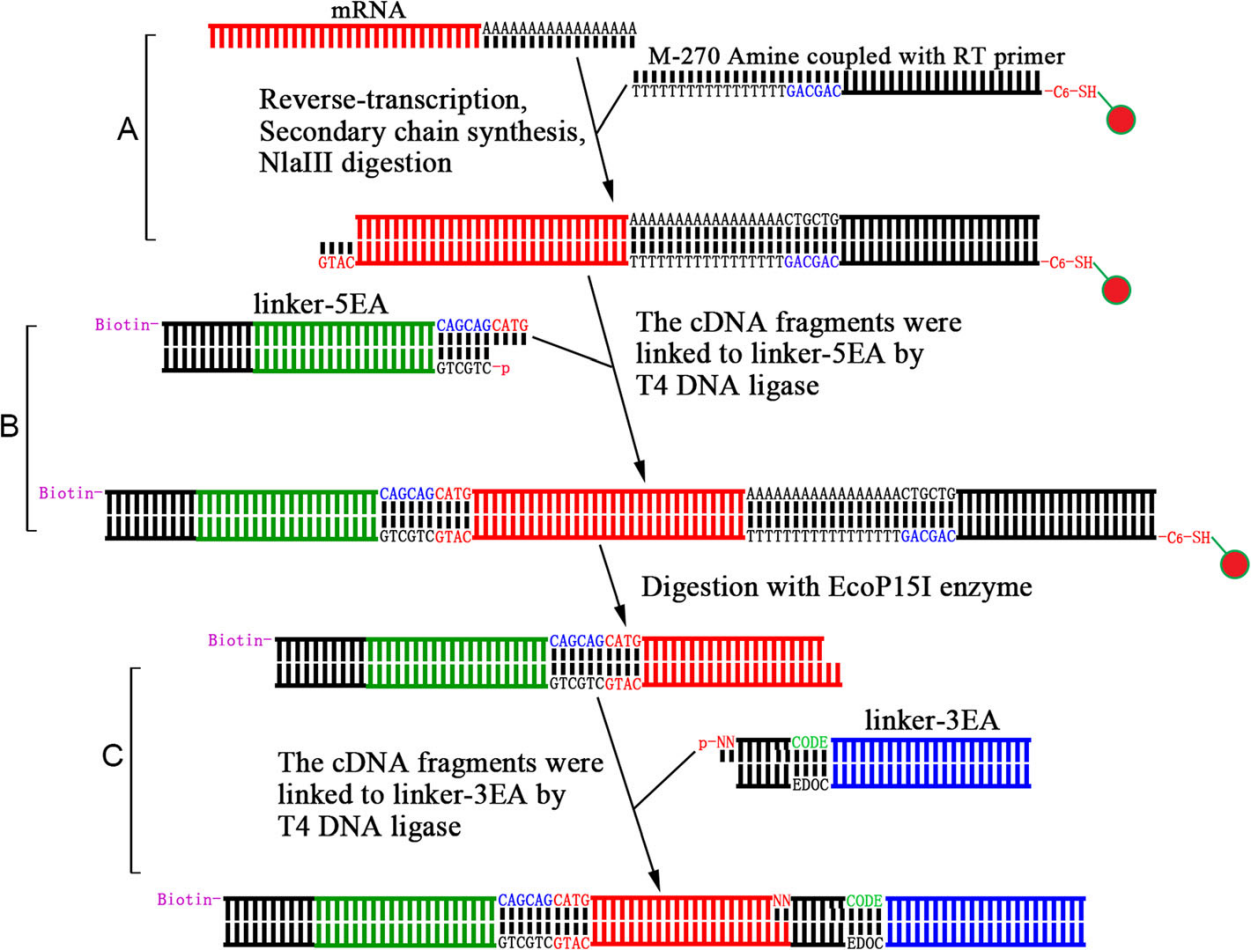
Flowchart of Super deepSAGE library construction. There are three major steps included in the protocol: **a**) reverse transcription with oligo (dT) coupled magnetic beads, synthesis of the secondary chain, and digestion with NlaIII; **b**) add 5′ end linker and digest with EcoP15I, and **c**) add 3′ end linker and PCR amplification. For details see materials and methods section

A flowchart of the Super deepSAGE experiment is summarized in Fig. 8. Dynabeads® M^− 270^ Amine (Thermo Fisher Scientific, China) were coupled with –C6-SH labeled reverse transcription-primer with the sequence containing the 5′-CAGCAG-3′ recognition site of EcoP15I and an Oligo (dT) sequence at the 3′ end, intentionally designed to complement the poly(A) sequence of mRNAs (Synthesized by Sangon Biotech, China). The coupling procedure was carried out as outlined in the protocol reported by Hill and Mirkin [30] using the succinimidyl 4-(p-maleimidophenyl) butyrate (SMPB) crosslink reagent (Thermo scientific, Shanghai, China). Ten micrograms of mRNA were reverse-transcribed (cDNA synthesis system, Invitrogen) with the Oligo (dT) magnetic beads to generate single-stranded cDNA using the manufacturer protocol. The product was converted to double-stranded cDNA using random primers and then digested with NlaIII (NEB, Beijing, China). The biotin-labeled linkers (linker-5EA) with phosphorylated 5′ termini and 3′ end overhangs (5′-CATG-3′) contain the EcoP15I recognition site were prepared by annealing commercially synthesized oligonucleotides. The magnetic beads-bound cDNA was washed and bound to linker-5EA by T4 DNA ligase (NEB, Beijing, China). As a result, each cDNA fragment bound to the magnetic beads was flanked by two inverted repeats of EcoP15I recognizing sites. The type III restriction enzyme EcoP15I cleaves the DNA downstream of the recognizing site (25 nt in one strand and 27 nt in the other strand) leaving a 5′ end overhang of two bases [31, 32]. Linker-ligated cDNAs on the magnetic beads were digested with ten units of EcoP15I under conditions described previously [33]. The supernatant containing biotin-labeled fragments were added to streptavidin magnetic beads (Promega, Beijing, China), and the biotin-labeled fragments of the cDNA were captured. Finally, barcoded linkers (linker-3EA) with two random base overhangs at 5′ end and phosphorylated termini were prepared and ligated to the cDNA ends by T4 DNA ligase (NEB, Beijing, China). The resulting products were amplified by polymerase chain reaction (PCR), and the 119 bp product was separated by polyacrylamide gel electrophoresis (PAGE) and recovered from the gel. The barcoded libraries prepared from different samples were combined into a single multiplex sequencing reaction at the end of library construction and submitted for deep sequencing. The sequence information of synthetic oligos, linkers, and primers are available in Supplemental document 4.

The serial analysis of gene expression (SAGE) was first developed by Velculescu et al. [34] and improved by Saha et al. [35], Matsumura et al. [33], and Nielsen et al. [36]. The traditional SAGE library construction protocol includes multiple steps, and the separation of the linker-tag fragment is challenging to perform, and the PAGE purification often produces low yield. The library construction protocol in this study was improved by introducing two magnetic beads: 1) Dynabeads® M^− 270^ Amine coupled with –C6-SH labeled Oligo (dT) reverse transcription primer; 2) The streptavidin magnetic beads which can capture biotin-labeled linkers (linker-5EA). The magnetic beads used in this protocol can capture and purify the DNA fragments and is technically less demanding than PAGE separation. This modification increased the yield of linker-tag fragments and resulted in the robustness of the technique. Also, the primers and linkers were designed compatible with multiplexed deep sequencing technology, eliminating additional sequencing costs.

### Animals, samples collection, and deep sequencing

Tissue samples were collected from a private slaughter farm (Jingzhou) located in the Hubei province in China. Following anesthesia by electric shock, specimens were excised, snap-frozen in liquid nitrogen, and kept in a deep freezer (− 80 °C) until RNA extraction. The sample collection was approved by the Animal Care and Use Committee of Hubei Province (China, YZU-2018-0031). RNA isolation and purification from tissues and cells was done using the RNeasy Mini Kit (Qiagen, Shanghai, China) following the manufacturer’s protocols. The BioAnalyzer 2100 (Agilent) was used to assess the integrity of total RNAs, and RIN number of less than 7.0 was eliminated from the study. After the experiment, the animals were slaughtered and sold. A total of 224 tissue samples across 28 different tissues were collected. The samples were collected from 32 animals from a Duroc × Landrace × Yorkshire (DLY) commercial crossbreed pig populations consisting of 16 males and 16 females at weaning dates of 21 days. The endometrium, placenta, and conceptus were collected from Landrace × Yorkshire (LY) sows of 12 days of gestation. Bone-marrow-derived macrophages were obtained by culturing bone marrow cells for 5–7 d in the presence of CSF-1 (0.5 ng/ml), as described previously for mouse macrophages [37]. Monocyte-derived macrophages were prepared according to a method published by Gao Y. et al. [38]. Monocyte-derived macrophages were cultured from CD14^+^ blood monocytes and polarization towards macrophage phenotypes was achieved by treating with M-CSF following a protocol published by Jaguin M. et al. [39]. The detailed sample information is summarized in Table 1 and Supplemental document 5. A total of eight samples (four male and four female) for each tissue were randomly selected form the 16 animals and submitted for deep sequencing. The computational extraction of tags from sequence data by the program (in-house designed) removes the two bases at the 5′ end. This ‘digital removal’ is performed to minimize the less accurate effect of two random bases, at the 5′ end of linker-3EA, and could potentially reduce the length of the tags, and affect the representative ability of the data. However, a direct link with a linker that has two random bases at the 5′ end forming overhangs will 1) enhance the efficiency of the link assay, and 2) no additional blunt ending process was needed to be compared with traditional SAGE method. These two bases were removed by the ‘reads filtering’ procedure, thereby lowering the systematic bias in the data.

Libraries were constructed using the Illumina TruSeq Small RNA Sample Prep Kit and were sequenced on an Illumina HiSeq2000 sequencer (50 bp and single-read) (Illumina Inc., USA) [40]. The sequencing data was filtered by a quality score (poor score < 0.5) for more than 20% of all the bases and then assessed using FastQC [41]. All the data discussed in this study have been deposited to the NCBI GEO database [42] under accession number GSE134461. Tag sequence was extracted, counted, and assigned for each transcript (*Sus scrofa* assembly 11.1, gene 99) using the SAGE software (modified to allow extraction of 19 bp tags), and then normalized for each sample by quantile normalization method [43]. For the tags assigned to multiple transcripts, the average copy numbers of those tags were used. The processed data is available to the reader in Supplemental document 5. The principal component analysis (PCA) was performed using the log_2_ tag counts of transcripts across all samples using statistical analysis with R software version 3.5. The tissue-specific transcripts were identified by comparing samples from each tissue to the overall tag count across all samples, and a threshold was set to fold change > 5.0, *p*-value < 1.0 × 10^− 6^ according to a method implemented in the limma package [44]. The limma mode used was “model.matrix(~ 0+factor(c (target_tissue, other_tissue)))”. Clustering analysis was performed by first using the K-means clustering method to separate transcripts into big groups, and then using Hierarchical clustering to build the internal structure of the transcripts within the groups according to the method reported by Gu et al. [45, 46]. Global-seeding procedures of BF98 [47] have been introduced into the K-means clustering algorithm to improve the consistency and quality of clustering results. The BF98 method employed a bootstrap-type procedure to determine the initial seeds for the centers. Several subsamples (recommended *n* = 10) of the dataset were clustered using K-means. Each clustering operation produced a different candidate set of centroids from which a new data set was constructed. This dataset was clustered using K-means, and the centroids were chosen as the initial seeds. The optimal BF98 clustering result on the Super deepSAGE data was obtained by “visualization” of the result performed by using K = 10 and the number of subsamples S = 1000 after trying K from 5 to 28 and S from 20 to 1000. The “visualization” method is straightforward for determining the best parameter for the K-means clustering procedure, but when the K reached 10, definite, compact and representative gene clustering was formulated, and when the S is higher than 200, consistent clustering results were produced for each duplicated clustering run.

### Luciferase reporter assay

A 1Kb nucleotide promoter segment of TLR-2, LCK, and VAV1 that included RUNX1 target sites was inserted upstream of a firefly luciferase ORF (pGL3, Promega, Beijing, China), and luciferase activity was compared to that of an analogous reporter with point substitutions disrupting the target sites, or analogous reporter with the binding site deleted completely. The logic behind the luciferase reporter assay is that deletion/mutation of a RUNX1 binding site should allow the down-regulation of its target genes, and hence the target gene should be expressed differently between the wild type and mutated constructs. The pGL3-Control activity was used for the normalization of firefly luciferase activity. For the assay, the cells were plated in a 96-well plate at 3000 cells per well. After overnight incubation, the cells were treated with a transfection reagent mixture consisting of 35 μL of serum-free medium, 0.3 μL of TransFast™ Transfection Reagent (Cat. E2431), and 0.02 μg of pGL3 and pGL3-Control vector per well. After a one-hour incubation, 100 μL of the serum-containing medium was added to the wells. 24 to 48 h post-transfection, EnduRen™ Live Cell Substrate (Cat. E6481) was added at a final concentration of 60 μM, and luciferase activity was monitored.

### PBMC isolation and stimulation

Peripheral blood mononuclear cells (PBMCs) were isolated from whole blood collected from five animals aged 21 days using BD Vacutainer^VR^ Cell Preparation Tubes (Becton Dickinson, Shanghai, China). The samples were processed according to the manufacturer’s instructions within two hours of blood collection. PBMCs were harvested from the tube, washed with phosphate-buffered saline (Life Technologies), and centrifuged for 10 min at 300 g before use. To induce gene expression, PBMCs were resuspended in RPMI-1640 medium (Life Technologies) supplemented with 10% fetal bovine serum (Life Technologies) at 1.5 × 10^6^ cells/mL in a 96-well V-bottom polypropylene plate (Corning Incorporated). LPS (Sigma-Aldrich, Shanghai, China) and RUNX1 inhibitor (Ro 5–3335, R&D Systems, Shanghai, China) were added at 5 ng/mL and 10 ng/mL, respectively, according to the manufacturer’s instructions. Untreated PBMCs were used as control samples.

### Surface staining and cytometry acquisition

Phenotypic surface staining was performed in BD Pharmingen™ stain buffer (BSA, BD Biosciences, Shanghai, China) for 30 min at room temperature in the dark, using anti-CD14 PE (BD Biosciences, Shanghai, China). Cells were washed and suspended in BD Pharmingen stain buffer (BSA, BD Biosciences, Shanghai, China), anti-TLR-2 FITC, anti-LCK FITC, anti-VAV1 FITC (BD Biosciences, Shanghai, China), was then added separately, and the mixture was incubated for 20 min at room temperature. Finally, cells were washed and acquired on a BD LSRFortessa™ cell analyzer (BD Biosciences, Shanghai, China). The flow cytometry data was deposited in the Flow Repository database [48] under accession number FR-FCM-Z268.

## Supplementary information

**Additional file 1.** Supplemental document 1 Sequencing matrix, the information of total number of reads, sequencing depth, coverage, and etc.

**Additional file 2.** Supplemental document 2 Clustering information, the detailed clustering information of tissue-differentially expressed genes.

**Additional file 3.** Supplemental document 3 RUNX1 targets, the 33 RUNX1 target genes that have RUNX1 binding site from Class 5.

**Additional file 4.** Supplemental document 4 Sequences and primers, the sequence information of synthetic oligos, linkers, and primers.

**Additional file 5.** Supplemental document 5 Sample information, the detailed sample information used in this study.

**Additional file 6.**

## Data Availability

The datasets generated and analyzed during the current study are available in the NCBI GEO (GSE134461) and Flow Repository database (FR-FCM-Z268). https://www.ncbi.nlm.nih.gov/geo/query/acc.cgi?acc=GSE134461 http://flowrepository.org/id/RvFrphtLijqf34kFNTA1gdB6BdXEskSDTdhZ4VwfM1qbgTIPfmqbL8o5eVTIhiUH

## Abbreviations

deepSAGE: Serial analysis of gene expression by deep sequencing
DLY: Duroc × Landrace × Yorkshire
EST: Expressed Sequence Tag
LCK: Lymphocyte-specific protein tyrosine kinase
LY: Landrace × Yorkshire
PAGE: Polyacrylamide gel electrophoresis
PAMPs: Pathogen-associated molecular pattern
PBMCs: Peripheral blood mononuclear cells
PCA: Principal component analysis
PCR: Polymerase chain reaction
RUNX1: Runt-related transcription factor 1
SAGE: Serial analysis of gene expression
SMPB: Succinimidyl 4-(p-maleimidophenyl)butyrate
TLR2: Toll-like receptor 2
VAV1: Vav1 oncogene

## References

[1] Verma N, Rettenmeier AW, Schmitz-Spanke S (2011). Recent advances in the use of Sus scrofa (pig) as a model system for proteomic studies. Proteomics.

[2] Houpt KA, Houpt TR, Pond WG (1979). The pig as a model for the study of obesity and of control of food intake: a review. Yale J Biol Med.

[3] Bailey KL, Carlson MA (2019). Porcine models of pancreatic cancer. Front Oncol.

[4] Schroyen M, Tuggle CK (2015). Current transcriptomics in pig immunity research. Mamm Genome.

[5] Groenen MA, Archibald AL, Uenishi H, Tuggle CK, Takeuchi Y, Rothschild MF, Rogel-Gaillard C, Park C, Milan D, Megens HJ (2012). Analyses of pig genomes provide insight into porcine demography and evolution. Nature.

[6] Beiki H, Liu H, Huang J, Manchanda N, Nonneman D, Smith TPL, Reecy JM, Tuggle CK (2019). Improved annotation of the domestic pig genome through integration of Iso-Seq and RNA-seq data. BMC Genomics.

[7] Dawson HD, Loveland JE, Pascal G, Gilbert JG, Uenishi H, Mann KM, Sang Y, Zhang J, Carvalho-Silva D, Hunt T (2013). Structural and functional annotation of the porcine immunome. BMC Genomics.

[8] Hornshoj H, Conley LN, Hedegaard J, Sorensen P, Panitz F, Bendixen C (2007). Microarray expression profiles of 20.000 genes across 23 healthy porcine tissues. PLoS One.

[9] Haverty PM, Weng Z, Best NL, Auerbach KR, Hsiao LL, Jensen RV, Gullans SR (2002). HugeIndex: a database with visualization tools for high-density oligonucleotide array data from normal human tissues. Nucleic Acids Res.

[10] Shmueli O, Horn-Saban S, Chalifa-Caspi V, Shmoish M, Ophir R, Benjamin-Rodrig H, Safran M, Domany E, Lancet D (2003). GeneNote: whole genome expression profiles in normal human tissues. C R Biol.

[11] Su AI, Cooke MP, Ching KA, Hakak Y, Walker JR, Wiltshire T, Orth AP, Vega RG, Sapinoso LM, Moqrich A (2002). Large-scale analysis of the human and mouse transcriptomes. Proc Natl Acad Sci U S A.

[12] Su AI, Wiltshire T, Batalov S, Lapp H, Ching KA, Block D, Zhang J, Soden R, Hayakawa M, Kreiman G (2004). A gene atlas of the mouse and human protein-encoding transcriptomes. Proc Natl Acad Sci U S A.

[13] Walker JR, Su AI, Self DW, Hogenesch JB, Lapp H, Maier R, Hoyer D, Bilbe G (2004). Applications of a rat multiple tissue gene expression data set. Genome Res.

[14] Freeman TC, Ivens A, Baillie JK, Beraldi D, Barnett MW, Dorward D, Downing A, Fairbairn L, Kapetanovic R, Raza S (2012). A gene expression atlas of the domestic pig. BMC Biol.

[15] Tang Z, Li Y, Wan P, Li X, Zhao S, Liu B, Fan B, Zhu M, Yu M, Li K (2007). LongSAGE analysis of skeletal muscle at three prenatal stages in Tongcheng and landrace pigs. Genome Biol.

[16] Wang B, Regulski M, Tseng E, Olson A, Goodwin S, McCombie WR, Ware D (2018). A comparative transcriptional landscape of maize and sorghum obtained by single-molecule sequencing. Genome Res.

[17] Son CG, Bilke S, Davis S, Greer BT, Wei JS, Whiteford CC, Chen QR, Cenacchi N, Khan J (2005). Database of mRNA gene expression profiles of multiple human organs. Genome Res.

[18] Frith MC, Fu Y, Yu L, Chen JF, Hansen U, Weng Z (2004). Detection of functional DNA motifs via statistical over-representation. Nucleic Acids Res.

[19] Khan A, Fornes O, Stigliani A, Gheorghe M, Castro-Mondragon JA, van der Lee R, Bessy A, Cheneby J, Kulkarni SR, Tan G (2018). JASPAR 2018: update of the open-access database of transcription factor binding profiles and its web framework. Nucleic Acids Res.

[20] Kasprzyk A (2011). BioMart: driving a paradigm change in biological data management. Database (Oxford).

[21] Wong WF, Kurokawa M, Satake M, Kohu K (2011). Down-regulation of Runx1 expression by TCR signal involves an autoregulatory mechanism and contributes to IL-2 production. J Biol Chem.

[22] Uchida H, Zhang J, Nimer SD (1997). AML1A and AML1B can transactivate the human IL-3 promoter. J Immunol.

[23] Zhang DE, Hetherington CJ, Meyers S, Rhoades KL, Larson CJ, Chen HM, Hiebert SW, Tenen DG (1996). CCAAT enhancer-binding protein (C/EBP) and AML1 (CBF alpha2) synergistically activate the macrophage colony-stimulating factor receptor promoter. Mol Cell Biol.

[24] Frank R, Zhang J, Uchida H, Meyers S, Hiebert SW, Nimer SD (1995). The AML1/ETO fusion protein blocks transactivation of the GM-CSF promoter by AML1B. Oncogene.

[25] Taniuchi I, Osato M, Egawa T, Sunshine MJ, Bae SC, Komori T, Ito Y, Littman DR (2002). Differential requirements for Runx proteins in CD4 repression and epigenetic silencing during T lymphocyte development. Cell.

[26] Medzhitov R (2001). Toll-like receptors and innate immunity. Nat Rev Immunol.

[27] Davis SJ, van der Merwe PA (2011). Lck and the nature of the T cell receptor trigger. Trends Immunol.

[28] DeFranco AL (2001). Vav and the B cell signalosome. Nat Immunol.

[29] Helou YA, Petrashen AP, Salomon AR (2015). Vav1 regulates T-cell activation through a feedback mechanism and crosstalk between the T-cell receptor and CD28. J Proteome Res.

[30] Hill HD, Mirkin CA (2006). The bio-barcode assay for the detection of protein and nucleic acid targets using DTT-induced ligand exchange. Nat Protoc.

[31] Moncke-Buchner E, Rothenberg M, Reich S, Wagenfuhr K, Matsumura H, Terauchi R, Kruger DH, Reuter M (2009). Functional characterization and modulation of the DNA cleavage efficiency of type III restriction endonuclease EcoP15I in its interaction with two sites in the DNA target. J Mol Biol.

[32] Meisel A, Bickle TA, Kruger DH, Schroeder C (1992). Type III restriction enzymes need two inversely oriented recognition sites for DNA cleavage. Nature.

[33] Matsumura H, Reich S, Ito A, Saitoh H, Kamoun S, Winter P, Kahl G, Reuter M, Kruger DH, Terauchi R (2003). Gene expression analysis of plant host-pathogen interactions by SuperSAGE. Proc Natl Acad Sci U S A.

[34] Velculescu VE, Zhang L, Vogelstein B, Kinzler KW (1995). Serial analysis of gene expression. Science.

[35] Saha S, Sparks AB, Rago C, Akmaev V, Wang CJ, Vogelstein B, Kinzler KW, Velculescu VE (2002). Using the transcriptome to annotate the genome. Nat Biotechnol.

[36] Nielsen KL, Hogh AL, Emmersen J (2006). DeepSAGE--digital transcriptomics with high sensitivity, simple experimental protocol and multiplexing of samples. Nucleic Acids Res.

[37] Sester DP, Beasley SJ, Sweet MJ, Fowles LF, Cronau SL, Stacey KJ, Hume DA (1999). Bacterial/CpG DNA down-modulates colony stimulating factor-1 receptor surface expression on murine bone marrow-derived macrophages with concomitant growth arrest and factor-independent survival. J Immunol.

[38] Gao Y, Flori L, Lecardonnel J, Esquerre D, Hu ZL, Teillaud A, Lemonnier G, Lefevre F, Oswald IP, Rogel-Gaillard C (2010). Transcriptome analysis of porcine PBMCs after in vitro stimulation by LPS or PMA/ionomycin using an expression array targeting the pig immune response. BMC Genomics.

[39] Jaguin M, Houlbert N, Fardel O, Lecureur V (2013). Polarization profiles of human M-CSF-generated macrophages and comparison of M1-markers in classically activated macrophages from GM-CSF and M-CSF origin. Cell Immunol.

[40] Eminaga S, Christodoulou DC, Vigneault F, Church GM, Seidman JG: Quantification of microRNA expression with next-generation sequencing. Current protocols in molecular biology 2013, Chapter 4:Unit 4 17. 10.1002/0471142727.mb0417s103 PMC4138881 23821442

[41] Wingett SW, Andrews S (2018). FastQ Screen: A tool for multi-genome mapping and quality control. F1000Res.

[42] Edgar R, Domrachev M, Lash AE (2002). Gene expression omnibus: NCBI gene expression and hybridization array data repository. Nucleic Acids Res.

[43] Pan M, Zhang J (2018). Quantile normalization for combining gene-expression datasets. Biotechnology & Biotechnological Equipment.

[44] Ritchie ME, Phipson B, Wu D, Hu Y, Law CW, Shi W, Smyth GK (2015). limma powers differential expression analyses for RNA-sequencing and microarray studies. Nucleic Acids Res.

[45] Gu Z, Eils R, Schlesner M (2016). Complex heatmaps reveal patterns and correlations in multidimensional genomic data. Bioinformatics.

[46] Yao M, Wu QH, Li J, Huang TH. K-walks: clustering gene-expression data using a K-means clustering algorithm optimised by random walks. Int J Data Min Bioinform. 2016;16(2):121.

[47] Bradley PS, Fayyad UM (1998). Refining initial points for K-Means clustering. Proc 15th International Conf on Machine Learning.

[48] Spidlen J, Breuer K, Rosenberg C, Kotecha N, Brinkman RR (2012). FlowRepository: a resource of annotated flow cytometry datasets associated with peer-reviewed publications. Cytometry A.

