# Supplementary material for: A transcriptional landscape of 28 porcine tissues obtained by super deepSAGE sequencing
Source: BMC Genomics. 2020 Mar 14;21:229. doi: 10.1186/s12864-020-6628-7 (PMC7071599; doi:10.1186/s12864-020-6628-7)
Supplement: Supplementary file 4 — Additional file 4. Supplemental document 4 Sequences and primers, the sequence information of synthetic oligos, linkers, and primers. [file 12864_2020_6628_MOESM4_ESM.docx]

| Name | Sequence (5’ to 3’) |
| --- | --- |
| RT-Primer | MB-SH-C6-5’-CTGATCTAGAGGTACCGGATCCCAGCAGTTTTTTTTTTTTTTTTT-3’ |
| Product-NlaIII | MB-SH-C6-5’-CTGATCTAGAGGTACCGGATCCCAGCAGTTTTTTTTTTTTTTTTTN_x_CATG-3’  \|\|\|\|\|\|\|\|\|\|\|\|\|\|\|\|\|\|\|\|\|\|\|\|\|\|\|\|\|\|\|\|\|\|\|\|\|\|\|\|\|\|\|\|\|\|  3’-GACTAGATCTCCATGGCCTAGGGTCGTCAAAAAAAAAAAAAAAAAN_x_-5’ |
| Linker-5EA | Biotin-5’-AATGATACGGCGACCACCGAGATCTACACTCTTTCCCTACACGACGCTCTTCCGATCTCAGCAGCATG-3’  p-5’-CTGCTGAGATCGGAAGAGCGTCGTGTAGGGAAAGAGTGTAGATCTCGGTGGTCGCCGTATCATT-3’ |
| Product-EcoP15I | FITC-5’-PL-TCTTTCCCTACACGACGCTCTTCCGATCTCAGCAGCATGN_21_-3’  \|\|\|\|\|\|\|\|\|\|\|\|\|\|\|\|\|\|\|\|\|\|\|\|\|\|\|\|\|\|\|\|\|\|\|\|\|\|\|\|  NH_2_ -3’-LP-AGAAAGGGATGTGCTGCGAGAAGGCTAGAGTCGTCGTACN_21_NN-5’ |
| Linker-3EA | p-5’-NNCGTGATCODEATCTCGTATGCCGTCTTCTGCTTG -3’  \|\|\|\|\|\|\|\|\|\|\|\|\|\|\|\|\|\|\|\|\|\|\|\|\|\|\|\|\|\|\|\|\|\|  3’-GCACTAEDOCTAGAGCATACGGCAGAAGACGAAC -5’ |
| Primer-L | 5’-AATGATACGGCGACCACCGAGA-3’ |
| Primer-R | 5’-CAAGCAGAAGACGGCATACGAG-3’ |
